# Analysis of *FsTyDC1* Gene from *Forsythia suspensa* in Response to Drought and Salt Stress Treatment

**DOI:** 10.3390/metabo15090628

**Published:** 2025-09-19

**Authors:** Jiaqi Xu, Jiaxi Chen, Meng Yuan, Panpan Wang, Wenwen Li, Yilong Li, Chong Yang, Shufang Lv, Zhanqiang Ma, Hongxiao Zhang, Huawei Xu, Xingli Zhao, Ting Wang, Dianyun Hou

**Affiliations:** 1 College of Agriculture, Henan University of Science and Technology, Luoyang 471023, China; 2 Henan Engineering Research Center for Evaluation and Innovative Utilization of Homology of Medicine and Food, Luoyang 471023, China; 3 State Key Laboratory of Crop Stress Adaptation and Improvement, Henan University, Zhengzhou 450046, China

**Keywords:** *FsTyDC1*, *Forsythia suspensa*, drought, salt stress, treatment

## Abstract

**Background**: *Forsythia suspensa* (Thunb.) Vahl is a perennial deciduous shrub of the Oleaceae family. Its dried mature fruits are used as medicine and hold an important position in traditional Chinese medicine. Tyrosine decarboxylase (TyDC) is a key enzyme involved in the synthesis of dopamine in Forsythia suspensa. At the same time, it also affects the growth and development of this species under biotic stress. **Methods**: This study examined the expression and function of *FsTyDC1* under drought and salt stress. The *TyDC* gene identified in *F. suspensa*, termed *FsTyDC1*, has an open reading frame (ORF) of 1518 bp. **Results**: qRT-PCR and subcellular localization analyses indicated that *FsTyDC1* is highly expressed in *F. suspensa* fruit and its protein is located in the cytoplasm. The gene was silenced using a pTRV2-FsPDS/*FsTyDC1* vector with virus-induced gene silencing. Following exposure to drought and salt stress, the leaves of *FsTyDC1*-silenced plants exhibited increased curling and wilting. **Conclusions**: The results indicate that *FsTyDC1* responds to both salt and drought stress, which provides a foundation for further investigation into the function of *FsTyDC1*.

## 1. Introduction

*Forsythia suspensa* is a deciduous shrub belonging to the Oleaceae family. In traditional medicine, it is widely used in China, Japan, Korea, and many European countries [1]. The fruit of *F. suspensa* contains a diverse array of chemical components, with lignans, flavonoids, terpenoids, and volatile oils being the main active ingredients [2]. Based on the different development periods and harvesting seasons, the fruits of *F. suspensa* can be categorized into ‘Qingqiao’ and ‘Laoqiao’, both of which have medicinal uses [3]. Reports indicate that the extract from *F. suspensa* fruit is effective in the treatment of diseases, such as anti-inflammatory, antibacterial, antioxidant, and neuroprotective properties, among others [4]. In recent years, drought, high temperatures, and biotic stress have been the main factors contributing to the decline in the yield of *F. suspensa*. For instance, in 2024, in the Shanxi region, due to the impact of a two-month-long drought and high-temperature weather, the yield of artificially cultivated *F. suspensa* decreased by approximately 40%, while the reduction for wild *F. suspensa* was even over 60%. Among them, drought stress is one of the most adverse abiotic stresses that hinders plants’ growth and productivity, threatening sustainable crop production and quality [5]. Drought will directly affect the absorption of nutrients by plants, increase water loss, slow down plant growth, and lead to a decrease in material accumulation and leaf pigment content [6]. Similarly, salt stress is a common challenge faced by plants. Crop loss due to salt stress is an increasing threat to agriculture worldwide [7]. It is of great significance to effectively counter the adverse effects of salt stress on the growth of plants [8]. To cope with salt stress, plants mainly close or shrink stomata on their aboveground segments, which helps reduce water and nutrient loss, thereby reducing the impact of salt stress [9,10].

In recent years, research on *F. suspensa* has mainly focused on its chemical composition [11,12], pharmacological activity [13], germplasm resources [14], and cultivation technology [15], with limited investigation of genes associated with stress resistance.

Numerous studies have shown that secondary metabolites play important roles in helping plants adapt to environmental stressors, which include dopamine (DA) [16], phloridzin [17], and myoinositol [18]. Among them, DA is a strong water-soluble antioxidant first isolated in plants, exhibiting higher anti-oxidative capabilities than catechin, glutathione, the flavonol quercetin, and the flavone luteolin [19]. As a catecholamine, DA is a biogenic amine characterized by a 3,4-dihydroxy-substituted benzene ring and is widely found in both plants and animals [20]. It is known that DA can alleviate the negative effects of salt stress on apple growth through its strong antioxidant activity and ability to maintain ion balance [21]. Moreover, DA can effectively enhance the resistance of crops to drought and low-nitrogen stress, and reduce the damage caused by low temperatures to apples and watermelons [22,23,24]. Similar to the biosynthetic pathways in animals [25], the biosynthetic pathway of catecholamines in plants involves the decarboxylation of tyrosine by TyDC to produce tyramine, followed by hydroxylation by mono-hydroxylase (MH) to DA [26]. To date, TyDC genes have been successfully cloned from a variety of species, including apple, poplar, pansy, and Chinese foxglove [27,28,29]. Research indicates that tyrosine decarboxylase enhances plant resistance to abiotic stresses, such as salinity, drought, and low nitrogen availability. Studies have shown that dark and red-light treatments inhibit TyDC activity [30].

Reports indicate that drought stress can significantly induce the expression of the TyDC gene in Arabidopsis and begonia. The increase in DA in the MdTyDC-transgenic plants alleviates the adverse effects of cadmium on plant growth and increases the activities of antioxidant enzymes, which reduces the level of ROS. This evidence demonstrates that TyDC plays a pivotal role in plant responses to both biotic and abiotic stresses [31]. VIGS is a method that utilizes the viral defense mechanisms of plants to suppress specific invasive viral transcripts. It is a powerful tool for studying the functions of plant genes, capable of silencing single or multiple members of a gene family, unlike traditional methods used for gene function analysis [32]. The viral vectors employed in VIGS fall into three main categories based on virus type: RNA, DNA, or satellite viruses, each with distinct characteristics and applications [33]. For example, using tobacco rattle virus (TRV) as a viral infection vector offers advantages such as prolonged silencing, high efficiency, and mild symptoms, making it the most widely used vector. In silencing assays, viral vectors are usually introduced into plants using Agrobacterium-mediated methods [34]. Currently, VIGS is widely used in many fields, such as the analysis of resilience in peppers [35], wheat [36], tomato, etc. [37]. The VIGS vector of the SlPR1b gene in tomato was constructed and transformed into a resistance material of tomato to explore the resistance of SlPR1b gene-silenced tomato in ralstonia solanacearum stress conditions [37]. The results indicated that SlPR1b had a good effect on tomato bacterial wilt resistance [37]. The silencing of LoMYB65 using VIGS, which may be related to lily pollen synthesis, affects the normal expression of lily pollen, leading to abnormal pollen development and reduced pollen volume [38]. A dwarfed phenotype was observed in GmAGB1s-silenced soybean plants, which indicated that GmAGB1s play an important role in soybean growth and development [39]. According to that report, silencing GmAGB1s compromised the resistance of soybean plants against Xanthomonas campestris pv. glycinea (Xag). The results showed that GmAGB1s play a positive regulatory role in soybean defense [39].

To date, there have been no reports on the role of TyDC in the stress response in *F. suspensa*. In the present study, the *FsTyDC1* gene was identified from transcriptomic data, and its function in the plant’s response to drought and salt stress was investigated. The findings provide a theoretical basis for further studies on stress resistance in *F. suspensa*.

## 2. Materials and Methods

### 2.1. Plant Materials and Treatment

The materials of *F. suspensa* were planted on the farm of Henan University of Science and Technology (N: 34.41, E: 112.27, Luoyang, China). The average temperature on the farm is 18 °C, which was suitable for *F. suspensa* growth. For each treatment of drought and salt stress, three *F. suspensa* materials with the same growth and characteristics were randomly selected. For the subcellular localization tests, Nicotiana benthamiana was utilized, which was grown in climatic chambers maintained at a temperature of (22 ± 2) °C and 60% humidity, following a 16 h/8 h light/dark regime, with light intensities ranging from 200 to 300 μmol m^−2^ s^−1^. When the new shoots had 2–3 pairs of leaves, they were used for experiments. We ensured proper water and fertilizer management for the plants, as well as routine pest and disease control. Callus tissues were treated with 400 mmol/L mannitol and 200 mmol/L NaCl [40]. The leaves of *F. suspensa*, which had been silenced, were subjected to drought treatment using a 20% PEG6000 spray [41], and salt stress was induced by watering with 4% NaCl [42]. Samples were collected and analyzed after 15–20 days.

### 2.2. Bioinformatics Analysis of FsTyDC1

The TyDC sequences were identified from transcriptomic sequence data, and ORFs were analyzed using the ORF Finder (https://www.ncbi.nlm.nih.gov/orffinder/, accessed on 1 June 2024) to obtain the full-length sequence of the *F. suspensa* TyDC gene. The *Arabidopsis* genome was obtained from TAIR (https://www.arabidopsis.org/, accessed on 1 June 2024). The TyDC gene sequences of related plants were queried using NCBI BLAST (version 2.17.0; https://blast.ncbi.nlm.nih.gov/, accessed on 1 June 2024), and a phylogenetic tree was constructed using MEGA 7.0 and named *FsTyDC1*. The physicochemical properties of the *FsTyDC1* protein were analyzed using the Expasy ProtParam tool (https://web.expasy.org/protparam/, accessed on 1 June 2024). The secondary structure of the protein was analyzed using SOPMA (secondary structure prediction method), and the presence of a signal peptide was assessed using SignalP5.0 (https://services.Healthtech.dtu.dk/services/SignalP-5.0/, accessed on 1 June 2024).

### 2.3. Cloning of the FsTyDC1 Gene

The cDNA was prepared according to the method provided by Shanghai Yi Sheng Company. All the primers were designed using PrimerPremer 5.0 software (Table 1) and synthesized by Biological Bioengineering (Shanghai, China). The PCR amplification system included 25 µL of 12.5 × 3 G Taq Master Mix for PAGE (Red Dye), 1 µL of 2.5 M forward and reverse primer, respectively, 1 µL of cDNA, and 9.5 µL of sterile water. The conditions for amplifying *FsTyDC1* were 94 °C for 5 min, 33 cycles of 94 °C for 30 s, 56 °C for 30 s, extension at 72 °C for 2 min, and finally 72 °C for 5 min. After amplification, the correct band length was analyzed by 1% agarose gel electrophoresis. The PCR-amplified product *FsTyDC1* was purified using the FastPure Gel DNA ExtractionMini Kit (Vazyme Biotech Co., Ltd. Nanjing, China). *FsTyDC1* was ligated into the pMD-18T vector (TaKaRa) with a 10 µL reaction system, including 4.0 µL of *FsTyDC1*, 1.0 µL of pMD-18T, and 5.0 µL of Solution I. The reaction conditions were 16 °C for 30 min. The 5.0 µL of conversion product *FsTyDC1* was added to 50 µL of the Escherichia coli DH5α competent cells, and the mixture was left to stand at ice temperature for 30 min. The mixture was subjected to a heat shock at 42 °C for 45 s, then immediately placed in an ice bath for 3 min. An amount of 900 μL of liquid LB was added, and the mixture was incubated at 37 °C with a speed of 200 rpm for 60 min. Next, we collected the bacterial cells, discarded the supernatant of 800 μL, and uniformly spread them on the LB agar plate. We then incubated it at 37 °C for 12 to 16 h. The cells were propagated and the plasmids were extracted for the following experiments.

### 2.4. Analysis of the Gene Expression Patterns of FsTyDC1

RNA was extracted from the leaves and fruit of *F. suspensa*, which were extracted and reverse-transcribed to cDNA. The qRT-PCR amplification system included 1 µL of cDNA, 10 µL of 2×ChamQ Universal SYBR qPCR Master Mix (Vazyme Biotech Co., Ltd. Nanjing, China), and 0.5 µL of primers for qRT-PCR F/R, respectively, and 20 µL of ddH2O was added. The internal reference gene selected was FsUKN1. The reaction procedure of qRT-PCR was 94 °C for 30 s, 33 cycles of 95 °C for 10 s, and 60 °C for 30 s. Following PCR amplification, the relative expression of *FsTyDC1* was calculated using the 2^−∆∆Ct^ method with FsUKN1 as the reference gene [43,44]. Each reaction contained three replicates.

### 2.5. Analysis of Subcellular Localization

The subcellular localized recombinant vector pCAMBIA 1300-*FsTyDC1*-GFP was constructed by homologous recombination. Homologous primers containing the Kpn I and BamH I restriction sites were designed using CE Design software (V.1.04) (Table 1).

The pCAMBIA 1300-*FsTyDC1*-GFP recombinant plasmid, the pCAMBIA 1300-GFP empty plasmid, and the cytoplasmic marker pCAMBIA 1300-35S-UGPase-mcherry-NOS plasmid were, respectively, transformed into the Agrobacterium competent strain GV3101.

Mix equal amounts of cytoplasmic Agrobacterium infection solution containing the marker pCAMBIA 1300-35S-UGPase-mcherry-NOS and the infection solution containing pCAMBIA 1300-*FsTyDC1*-GFP and an empty vector Agrobacterium, respectively, and then infect tobacco. The tobacco leaves were inoculated by injecting the lower epidermis of the leaves with the tobacco extract. The specific method is as follows. Using a disposable sterile syringe, inject the 1 mL mixed infection solution into the tobacco leaves. When one observes that there are moist marks on the entire leaf, it indicates that the injection is complete, and the injection area should be marked. Under room temperature conditions, perform a 2-day dark cultivation to facilitate observation.

The protein subcellular localization was observed using a laser confocal microscope (Olympus, FV 3000). A small amount of distilled water was dropped onto the slide, and the leaf within the marked area was cut out. The surrounding injection holes were not damaged. The lower epidermis side of the leaf was placed face-up on the slide. The entire leaf was soaked in distilled water, and then a cover glass was placed on top to expel any air bubbles. The tobacco leaf was observed using the laser confocal microscope. 488 nm was the excitation wavelength for green fluorescent protein (GFP), and 583 nm was the excitation wavelength for the cell cytoplasmic marker protein with red fluorescent protein (RFP). The empty control and pCAMBIA 1300-*FsTyDC1*-GFP were observed at 488 nm, and UGPase-mcherry with the cell cytoplasmic marker was observed at 583 nm. The magnification we used was 400 times.

### 2.6. Construction of FsTyDC1 Gene Silencing Vector and Expression Analysis

Specific fragments of FsPDS and *FsTyDC1* (385 bp and 368 bp, respectively) were amplified through PCR. The oligonucleotide primers were synthesized by Sangon Biotech (Shanghai, China). The resulting products were cloned into pTRV2 to form TRV-FsPDS and pTRV2-*FsTyDC1* using the In-Fusion R HD Cloning Kit (TaKaRa Bio USA, Inc., San Jose, CA, USA). We mixed equal volumes of pTRV-FsPDS, pTRV-TyDC1 and pTRV2 with equal volumes of pTRV1, then let them stand in a dark environment at room temperature for 3 h before use. The leaves of *F. suspensa* were stained using the leaf injection method. After the injection was completed, the plants were cultured in the dark for 24 h and then cultivated normally. After about 15 to 20 days, the results were observed and measured. The testing methods referenced in the literature were followed in accordance with previously published protocols [45].

### 2.7. Statistical Analysis

The expression change and significance analysis of *FsTyDC1* in different developmental stages was performed using SPSS 5.0 software.

## 3. Results

### 3.1. Identification of TyDC from F. suspensa

**Figure 1:**
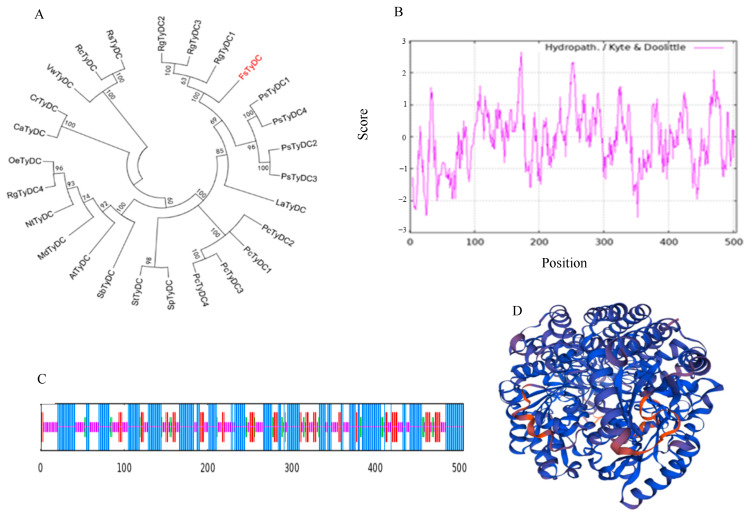
Analysis of the basic characteristics of *FsTyDC1*. (**A**) Phylogenetic tree of *FsTyDC1* in *F. suspensa* (the bootstrap scores (1000 replicates) are shown (≥50%) for each branch). (**B**) Conserved domain analysis of the *FsTyDC1* protein. (**B**) Hydrophobicity/hydrophilicity analysis of the *FsTyDC1* protein. (**C**) Secondary structure prediction of the *FsTyDC1* protein. (**D**) Three-dimensional structure prediction of the *FsTyDC1* protein. (Note: RgTyDC: *Rehmannia glutinosa* TyDC, FsTyDC: *Forsythia suspensa* TyDC, PsTyDC: *Papaver somn*; *iferum* TyDC, LaTyDC: *Lycoris aurea* TyDC, PcTyDC: *Petroselinum crispum* TyDC, StTyDC: *Solanum tuber*; *osum* TyDC, SbTyDC: *Sorghum bicolor* TyDC, StTyDC: *Solanum tuberosum* TyDC, AtTyDC: *Arabidopsis*; *thaliana* TyDC, MdTyDC: *Malus domestica* TyDC, NtTyDC: *Nicotiana tabacum* TyDC, OeTyDC: *Olea europaea* TyDC, CaTyDC: *Camptotheca acuminate* TyDC, CrTyDC: *Catharanthus roseus* TyDC, VwTyDC: *Viola×wittrockiana* TyDC, RcTyDC: *Rhodiola crenulate* TyDC, RsTyDC: *Rhodiola sachalinensis* TyDC).

In this study, *FsTyDC1* was identified from the *F. suspensa* genome by BLAST analysis. The protein sequence was used together with various TyDC sequences to construct a phylogenetic tree using MEGA 7.0 (Figure 1A). This analysis showed that *FsTyDC1*, RgTyDC1, RgTyDC2 and RgTyDC3 from *Rehmannia glutinosa* have united into one group. Among them, the *FsTyDC1* protein of *F. suspensa* is the most similar to RgTyDC1. This similarity was further confirmed by examining the structural domains of the protein. However, *FsTyDC1* has a very distant genetic relationship with MdTyDC, StTyDC, etc., and does not form a group together. The hydrophilicity of the protein is shown in Figure 1B, indicating that *FsTyDC1* is an unstable hydrophilic protein. The results of the secondary structure prediction are shown in Figure 1C, and the predicted model of the protein’s tertiary structure is shown in Figure 1D. The protein is predicted to contain 47.13% α-helix, 32.28% random coil, 14.85% β-sheet, and 5.74% β-turn.

### 3.2. Cloning and Vector Construction of FsTyDC1

**Figure 2:**
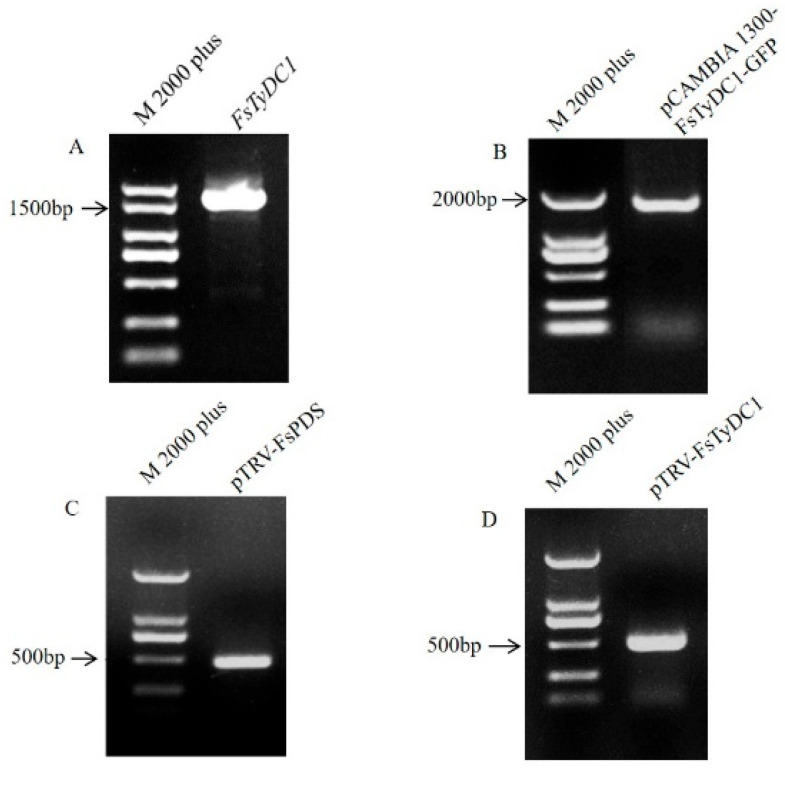
cDNA fragment of *FsTyDC1*. (**A**) cDNA sequence of *FsTyDC1*. (**B**) pCAMBIA 1300-*FsTyDC1*-GFP colony PCR. (**C**) pTRV-FsPDS colony PCR result. (**D**) pTRV-*FsTyDC1* colony PCR result.

The target fragments of *FsTyDC1* were amplified using PCR and examined using 1% agarose gel electrophoresis (Figure 2A). Sequence analysis confirmed that the cDNA sequence of the *FsTyDC1* was 1518 bp in length (GenBank No. OR725691). The pCAMBIA 1300-*FsTyDC1*-GFP (Figure 2B) recombinant expression vector and pTRV2-FsPDS/*FsTyDC1* vector (Figure 2C,D) were constructed and transferred into Agrobacterium sensu lato GV3101 and set aside for further use.

### 3.3. Spatiotemporal Expression Patterns and Drought and Salt Response of FsTyDC1

**Figure 3:**
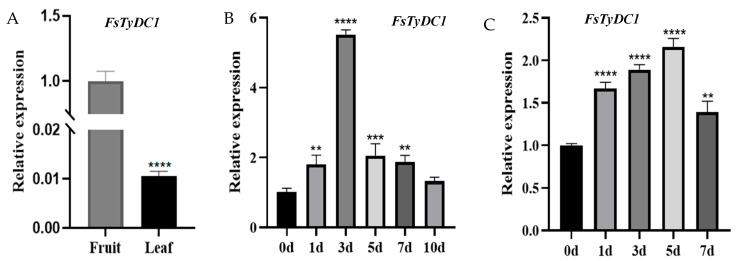
Expression analysis of *FsTyDC1* in different periods and tissues of *F. suspensa*. (**A**) Expression of *FsTyDC1* in leaves and fruits of *F. suspensa*. (**B**) Expression of *FsTyDC1* in different drought treatment times with 400 mmol/L mannitol. The expression of the *FsTyDC1* gene was extremely significant after 3 days of drought treatment. (**C**) Expression of *FsTyDC1* in different salt treatment times with 200 mmol/L NaCl. The expression of the *FsTyDC1* gene was extremely significant after 1, 3, and 5 days of salt treatment (Note: ** stands for *p* < 0.01, *** stands for *p* < 0.001, **** stands for *p* < 0.0001).

The relative expression levels of *FsTyDC1* genes in different tissues during different phases of drought and salt treatments were investigated using qRT-PCR in the leaves and fruits of *F. suspensa*. The induction of the expression levels of the target genes varied at different developmental stages, with *FsTyDC1* showing higher expression in fruits compared to leaves (Figure 3A).

After drought treatment, the expression of *FsTyDC1* initially increased and then decreased, reaching its maximum at 3 days of treatment (Figure 3B). After 5 days of salt treatment, the expression of *FsTyDC1* peaked, indicating that it responds to both drought and salt stress (Figure 3C).

### 3.4. Subcellular Localization Analysis of FsTyDC1 Proteins

**Figure 4:**
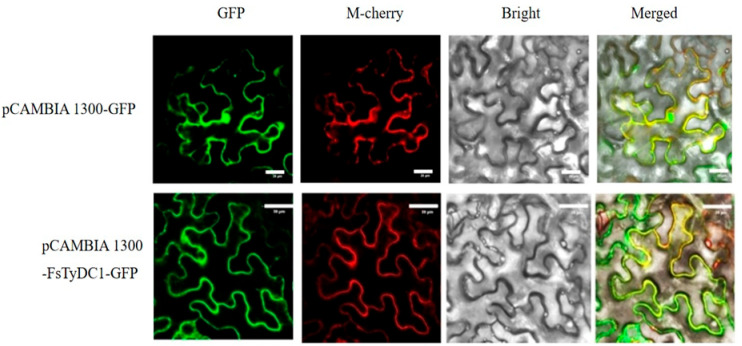
Subcellular localization of the *FsTyDC1* protein. “Green fluorescence” represents GFP fluorescence, “M-cherry” is a protein that is specifically located in the cytoplasm, “red fluorescence” represents cytoplasm, and “yellow fluorescence” represents the overlap of green and red fluorescence, demonstrating their co-location in the cytoplasm.

The recombinant expression vector pCAMBIA 1300-*FsTyDC1*-GFP was constructed and introduced into an Agrobacterium tumefaciens GV3101, which was then used to infect the lower epidermis of tobacco leaves. The results were observed using laser confocal microscopy (Figure 4). Using the pCAMBIA 1300-GFP empty vector and UGPase-mcherry as dual controls, under a laser confocal microscope, the fluorescence of the cytoplasmic Marker-labeled protein is red, the fluorescence of gene expression is green, and when the gene fluorescence and Marker fluorescence fully fuse, it presents yellow fluorescence, indicating correct localization. The results are shown in the figure. It was observed that the yellow fluorescence area represents the region where the marker and the gene overlap. That is, the fluorescence observed is the one emitted by the *FsTyDC1* protein and the one emitted by the cytoplasmic Marker protein, with overlapping areas. The complete expression fusion region in the intact tobacco leaf cells is shown, indicating that the *FsTyDC1* protein is expressed in the cytoplasm, which is consistent with the software prediction results.

### 3.5. Effects of FsTyDC1 Silencing on Drought and Stress Responses in F. suspensa

**Figure 5:**
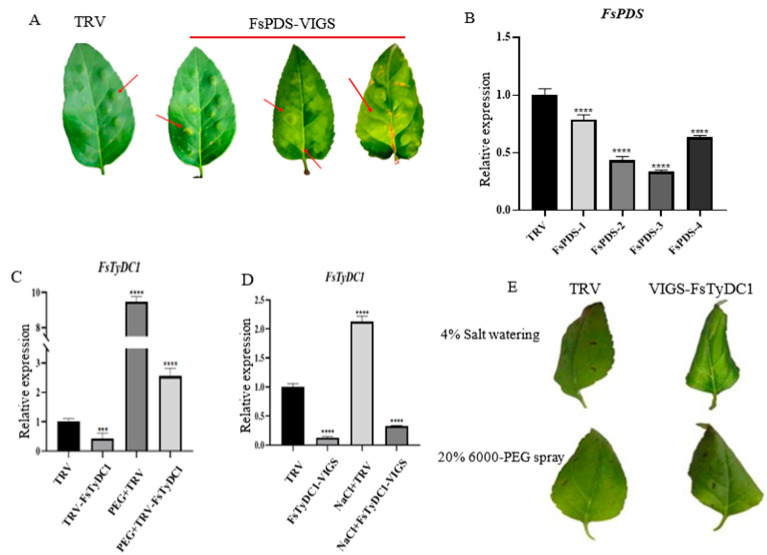
Establishment of the FsPDS silencing system and analysis of VIGS-*FsTyDC1* *F. suspensa* under drought and salt stresses. (**A**) Phenotypic changes in *F. suspensa* leaves treated with FsPDS-VIGS. The leaf surface was bleached near the pinhole injection, but there was no bleaching near the vacuum of the unloaded blade. The VIGS-FsPDS silencing system can play a normal silencing role in *F. suspensa* leaves. (**B**) FsPDS expression of leaves in *F. suspensa* after FsPDS-VIGS silencing. TRV was the control. FsPDS-1, FsPDS-2, FsPDS-3 and FsPDS-4 represented different silenced leaves, respectively. Compared with the control group, the relative expression of the FsPDS gene in photobleached plants decreased. (**C**) The *FsTyDC1* gene expression of *F. suspensa* leaves under PEG 6000 treatment. (**D**) The *FsTyDC1* gene expression of *F. suspensa* leaves under NaCl treatment. (**E**) The phenotype of *F. suspensa* leaves after *FsTyDC1* silencing. Note: *** stands for *p* < 0.001, **** stands for *p* < 0.0001.

The VIGS technique was used to study the response of *FsTyDC1* to drought and salt stress. The successful silencing was shown by the white coloration of *F. suspensa* leaves infected with pTRV2-PDS (Figure 5A). The silencing effect of FsPDS was found to exceed 50% at maximum, indicating the effectiveness of the gene silencing (Figure 5B).

The effects of drought and salt stress on plants with silenced *FsTyDC1* were studied through qRT-PCR. The results indicated that the expression of *FsTyDC1* was significantly downregulated in pTRV2-*FsTyDC1* plants (Figure 5C,D), indicating an increase in expression levels in both control and silenced plants. In addition, the leaves of *F. suspensa* were subjected to drought treatment. After 20 days of drought and salt stress treatment, the growth of pTRV2 plants was better than that of PTRV2-*FsTyDC1* plants. Furthermore, the gene-silenced plants showed a higher degree of wilting and leaves curling (Figure 5E), which are characteristic responses to drought. This suggests that pTRV2-*FsTyDC1* plants experienced significantly more stress than pTRV2 plants.

## 4. Discussion

Currently, research on abiotic stress in *F. suspensa* mainly focuses on comparisons among different varieties, with fewer studies addressing genes related to stress resistance. DA influences phytological growth and developmental processes by interacting with phytohormones, and plays a significant role in plant growth and responses to adversity [46]. TyDC is an important enzyme in the synthesis of dopamine, and findings indicate that TyDC enhances a plant’s resistance to various abiotic stresses, such as salinity, nutrient deficiency, drought and low nitrogen levels [47]. In recent years, there has been an increase in studies exploring the functions of TyDC genes in relation to stress resistance, providing valuable references and insights for our research [27,30,31]. However, comprehensive analyses of TyDC gene information in *F. suspensa* remain scarce. In this study, one FsTyDC gene was identified from the transcriptome sequences of F. suspensa. The protein phylogenetic tree showed that FsTyDC clustered in a small branch with the RgTyDC gene family of Rehmanniae Radix, which led to its designation as *FsTyDC1*. The gene was systematically analyzed regarding its physicochemical properties, gene structure, and protein structure. Predictions indicate that *FsTyDC1* is an unstable hydrophilic protein, which aligns with predictions made for Viola tricolor [48]. The *FsTyDC1* and Gloriosa superba GsTyDC1 genes exhibited tissue-specific expression [25]. The results showed that *FsTyDC1* expression levels were higher in fruits, which are the primary medicinal parts of *F. suspensa*.

Overexpression of MdTYDC improved drought tolerance in apple plants and increased dopamine content [44]. In addition, the response pattern under both salt and drought stress was analyzed, revealing that *FsTyDC1* expression was significantly up-regulated in response to both 400 mmol/L mannitol and 200 mmol/L NaCl treatments. This indicates that the *FsTyDC1* gene exhibits a responsive reaction to both drought and salt stress, aligning with the results of other studies. At the same time, it also indicates that, like other plants, *F. suspensa* has a much broader metabolic adaptability to the external environment [49].

In this study, the *FsTyDC1* gene was also affected by drought stress, with expression levels significantly differing between leaves and fruits under different durations. Under drought conditions, the expression of the *FsTyDC1* gene initially increased and then decreased, peaking on the third day of treatment. It is also possible that the *FsTyDC1* gene participates in ABA biosynthesis, similar to the CCD genes in *F. suspensa*.

In addition, comparing gene expression differences across different populations of *F. suspensa* under drought stress showed that the HBWZ population might exhibit higher drought tolerance among the four populations studied, which include Wulaofeng, Shanxi (SXWL), soluble sugar (SS), Wuzhi Mountains, Hebei (HBWZ) and Shaanxi Hua Mt. (SXHM). HBWZ showed the most significant growth after drought treatment, suggesting that different *F. suspensa* cultivars have different responses to drought stress [50]. This study focused solely on the *FsTyDC1* gene in *F. suspensa* from one region in Luoyang under drought stress. Further research is needed to investigate the expression of *FsTyDC1* in populations from other areas. Additionally, the study did not analyze the changes in related physiological indices of *F. suspensa* under drought stress.

Subcellular analysis showed that the *FsTyDC1* protein was localized in the cytoplasm, consistent with predicted results. This finding aligns with the localization patterns of *FsTyDC1* proteins in Gloriosa superba [36], Rehmanniae Radix [51], and Arabidopsis thaliana [52], suggesting that the *FsTyDC1* gene may possess functional characteristics similar to those of other plant homologs.

Studying the functions of stress resistance genes is one of the most practical applications of plant VIGS for identifying gene roles [53]. Chung [54] first successfully established and applied this technology by silencing the PDS gene in chili peppers, resulting in leaf albinism. In cotton [55], drought treatment revealed that plants with silenced GhVHA-A genes suffered greater injury. In this study, silencing FsPDS established a silencing system for forsythia leaves, leading to the suppression of gene expression in plants with silenced *FsTyDC1*. Under drought and salt stress conditions, leaves of *F. suspensa* with the silenced *FsTyDC1* gene showed increased curling and wrinkling, indicating that this gene reduced the plant’s tolerance to drought stress. In conclusion, the *FsTyDC1* gene in *F. suspensa* may play an important role in regulating plant tolerance to drought and salt stress.

## 5. Conclusions

In summary, bioinformatics and expression analyses showed that *FsTyDC1* responded to both salt and drought stress. These findings lay the groundwork for further functional studies on the role of this gene in the biosynthesis of glycosides in *F. suspensa*. The results also indicated that *FsTyDC1* was highly expressed in *F. suspensa* fruit in the cytoplasm and responded to both salt and drought stress. Further investigation is needed to explore the molecular mechanisms and potential functions of the FsTyDC gene.

## Figures and Tables

**Table 1:** *FsTyDC1* gene primers.

Primer Name	Primer Sequence (5′-3′)	Annealing Temperature (°C)	Product Sizes (bp)
*FsTyDC1*-F	ACCCTCATTCACAGGTAGCAA	56	1500
*FsTyDC1*-R	CAAAACACGATACAGCAAAGATT		
qPCR-FsUKN1-F	CAGACCAGCTTTGAGGAGTATC	60	90
qPCR-FsUKN1-R	GGCCAGAAACCAGTAGTCAATA		
qPCR-*FsTyDC1*-F	CCGAGCAGTCTCAACGACAA	60	108
qPCR-*FsTyDC1*-R	CGCAAAGAAATAATGGAACCAG		
GFP-*FsTyDC1*-F	cgggggactgagctcggtaccATGGAAACTACGACTCGATGCTC	56	1515
GFP-*FsTyDC1*-R	catgtcgactctagaggatccTAAGATAGCTTCTGGAAGTCTCGGT		
VIGS-*FsTyDC1*-F	gtgagtaaggttaccgaattcCACCATCGGAACCACGTCA	56	370
VIGS-*FsTyDC1*-R	cgtgagctcggtaccggatccATAGCTACGAAGCACCAGCCA		

## Data Availability

The datasets presented in this study can be found in online repositories. The sequencing data can be found in the National Center for Biotechnology Information (NCBI) Sequence Read Archive under accession number No. OR725691.

## Abbreviations

The following abbreviations are used in this manuscript:

TyDC	Tyrosine decarboxylase
qRT-PCR	Quantitative reverse transcription
ORF	Open reading frame
DA	Dopamine
ABA	Abscisic acid
ROS	Reactive oxygen species
VIGS	Virus-induced gene silencing
TRV	Tobacco rattle
